# Viral load of Torquetenovirus correlates with Sano’s score and levels of total bilirubin and aspartate aminotransferase in Kawasaki disease

**DOI:** 10.1038/s41598-023-45327-5

**Published:** 2023-10-21

**Authors:** Pietro Giorgio Spezia, Kazunari Matsudaira, Fabio Filippini, Takako Miyamura, Keiko Okada, Yoshiro Nagao, Takafumi Ishida, Tetsuya Sano, Mauro Pistello, Fabrizio Maggi, Junichi Hara

**Affiliations:** 1 Department of Translational Research, University of Pisa, Pisa, Italy; 2 Present Address: Laboratory of Virology, National Institute for Infectious Diseases “Lazzaro Spallanzani”, Rome, Italy; 3 Division of Southeast Asian Area Studies, Graduate School of Asian and African Area Studies, Kyoto University, Kyoto, Japan; 4 Department of Pediatrics, Osaka University Graduate School of Medicine, Osaka, Japan; 5 Department of Haematology/Oncology, Osaka City General Hospital, Osaka, Japan; 6 Present Address: Department of Paediatrics, Fukuoka Tokushukai Hospital, 4-5 Sugukita, Kasuga City, Fukuoka 816-0864 Japan; 7 Department of Biological Sciences, University of Tokyo, Tokyo, Japan; 8 Department of Paediatrics, Japan Health Care Organisation Osaka Hospital, Osaka, Japan; 9 Virology Unit, University Hospital of Pisa, Pisa, Italy

**Keywords:** Microbiology, Diseases, Medical research, Pathogenesis, Risk factors

## Abstract

Cause of Kawasaki disease (KD) is unknown. KD is often resistant to treatment with intravenous immunoglobulin (IVIG). Sano’s score, which is derived from total bilirubin (TBIL), aspartate aminotransferase (AST) and C-reactive protein (CRP), is predictive of IVIG resistance in Japan. A recent study reported that Torquetenovirus (TTV), especially TTV7, was present at a high viral load in the patients with KD. We used PCR to quantify TTV load and amplicon next generation sequencing to detect individual TTV species. We used serum samples that were collected between 2002 and 2005 from 57 Japanese KD patients before IVIG treatment. Correlations between TTV load and Sano’s score, the biomarkers that constitute this score, and IVIG resistance were examined. TTV load was positively correlated with Sano’s score (P = 0.0248), TBIL (P = 0.0004), and AST (P = 0.0385), but not with CRP (P = 0.6178). TTV load was marginally correlated with IVIG resistance (P = 0.1544). Presence of TTV7 was correlated with total TTV load significantly (P = 0.0231). The correlations between biomarkers for KD and TTV load suggested that TTV may play a role in the pathophysiology of KD. We hypothesize that TTV7 may be associated with a higher total viral load in KD.

## Introduction

Kawasaki disease (KD) is a febrile illness characterized by mucocutaneous manifestations (e.g., conjunctivitis, cheilitis, rash, cervical lymphadenopathy, and/or oedematous extremities)^1,2^. Unless the disease is diagnosed and treated promptly, 40% of children with KD may develop coronary aneurysms^3^, which can form thromboses or stenoses^4^. In Japan, 17,000 children are affected by KD annually. Nearly 400,000 Japanese children have suffered from this potentially lethal illness^5^. Increasing numbers of cases have been reported in other countries and regions.

Developed in the 1980s–1990s, intravenous immunoglobulin (IVIG) is the mainstay of treatment of KD^6,7^. However, even with IVIG treatment, 20–40% of patients with KD relapse and require additional immunomodulatory therapy^8,9^. Multiple scoring systems have been developed to predict this “IVIG resistance”. The scores developed by Sano^10^, Kobayashi^11^, and Egami^12^ are frequently used in Japan, although these are less sensitive when used in non-Japanese populations^13^. A patient with KD who is predicted to have a high risk of IVIG resistance will be given immunomodulatory therapy in conjunction with the first IVIG treatment. For example, using Sano’s scoring method, either total bilirubin (TBIL) ≥ 0.9 mg/dL, aspartate aminotransferase (AST) ≥ 200 IU/L, or C-reactive protein (CRP) ≥ 7 mg/dL constitutes one score. A summed score ≥ 2 indicates a high-risk patient to whom an intravenous methylprednisolone pulse dose would be given^10^. Even with this augmented treatment, a substantial proportion of those patients develop IVIG resistance. Therefore, more specific modes of diagnostic tests and treatments have been needed. However, the cause of KD remains unknown 60 years after its discovery^14^, thus hindering development of specific diagnostic tests or treatments.

Epidemiological characteristics of KD, such as the presence of seasonality or spatiotemporal clustering^15–17^, suggest that KD is triggered by an infectious agent. It is likely that a genetically predisposed child can develop KD when they are infected with a causal microbe^18–21^. Epidemiological comparisons between KD and paediatric infectious diseases support presence of a viral agent for KD^22,23^.

A prospective metagenomic study identified high viral loads of Torquetenovirus (TTV) 7 in some patients with KD, while this virus was not identified in control subjects^24^. Although the result was not statistically significant due to the small sample size, this finding warrants further study to examine the possible association between KD and TTV. Use of quantitative PCR technology has been increasingly common in the study of TTV kinetics^25^. Using this technology, we quantified the viral loads of TTV in serum samples from patients with KD. In addition, we used newly developed amplicon next generation sequence (NGS) to identify individual species of TTV. These virological data were correlated to biomarkers that constitute Sano’s score and to IVIG resistance.

## Results

### Participants

Between 2002 and 2005, 236 patients with KD were enrolled from four hospitals in Osaka prefecture (i.e. Itami city hospital, Ikeda municipal hospital, Toyonaka municipal hospital and Minoh city hospital). Per the study protocol, we stored the serum only after written informed consent was obtained from the patients’ guardians. We collected pre-treatment serum samples from a total of 61 patients. Four of these pre-treatment samples had insufficient volume (< 200 μL). Therefore, the analysis included serum samples collected from 57 patients (Supplementary File 1). Of these patients, pre-treatment serum samples were collected from six patients one day before the first IVIG treatment and from 51 patients on the day of the first IVIG treatment. We also analysed longitudinal samples collected from these patients between day − 1 and day 15. Of the 57 patients studied, 30 were male (53%). The mean age of the patients was 3.0 years (median: 2.7 years; range: 0.2–9.3 years). The first IVIG was given to all patients. The median timepoint of the first IVIG was day 4 of the course of the illness (range: days 2–11), assuming that the onset of KD is day 1. In seven patients, whether the patient was IVIG-resistant was not recorded. Among the remaining 50 patients, 8 (16%) were resistant to the first IVIG treatment. On the day of treatment initiation, Sano’s score indicated a high risk of IVIG resistance (i.e., ≥ 2 score value) in 17 patients (30%). These patients were given an intravenous methylprednisolone pulse on the first day of IVIG treatment.

### Correlation between biomarkers for KD and TTV viral load

**Figure 1:**
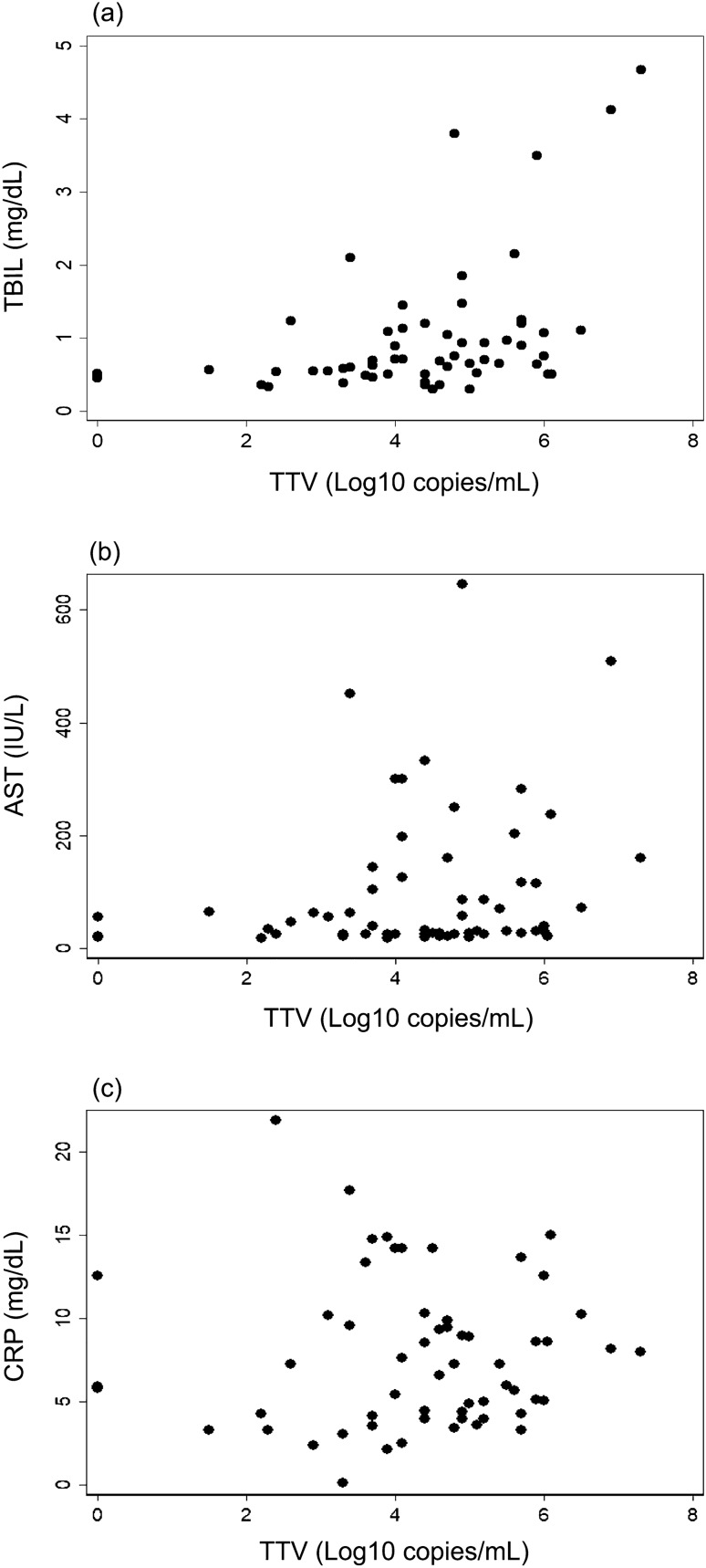
The biomarkers that constitute Sano’s score, and viral loads of Torquetenovirus (TTV). TTV viral loads were plotted against TBIL (**a**), AST (**b**), and CRP (**c**), which constitute Sano’s score that predicts IVIG resistance. TBIL and AST were positively correlated with TTV viral load; the result was statistically significant.

The TTV viral load was positively correlated with TBIL and AST, but not with CRP (Fig. 1). The highly skewed distributions of these biomarkers were normalised^26^, in subsequent statistical analyses (Supplementary File 2). Spearman’s rank correlation analysis (Table 1a), univariate linear regression analysis (Table 1b), and multivariate linear regression analysis (Table 1c) indicated that the correlations between TTV load and TBIL/AST were statistically significant (P < 0.05).

**Table 1:** Correlations between Torquetenovirus viral loads and biomarkers for Kawasaki disease.

n = 57	TBIL	AST	CRP
(a) Spearman’ rank correlation analysis			
TTV	R = 0.4476 (P = 0.0005)	R = 0.2743 (P = 0.0389)	R = 0.1114 (P = 0.4092)

(b) Univariate regression analysis^†^	Coefficients (P value)	Coefficients (P value)	Coefficients (P value)
TTV	0.192 (P = 0.0007)	0.0170 (P = 0.0412)	0.0604 (P = 0.6449)
Adjusted R^2^	0.176 (P = 0.0007)	0.0568 (P = 0.0412)	− 0.0142 (P = 0.6449)

(c) Multivariate regression analysis^†^	Coefficients (P value)	Coefficients (P value)	Coefficients (P value)
TTV	0.203 (P = 0.0004)	0.0174 (P = 0.0385)	0.0671 (P = 0.6178)
Sex (male 1; female: 0)	− 0.286 (P = 0.0748)	− 0.0262 (P = 0.3123)	0.0138 (P = 0.9738)
Age (years)	− 0.017 (P = 0.6958)	− 0.0065 (P = 0.3303)	0.0721 (P = 0.5068)
Adjusted R^2^	0.1973 (P = 0.0021)	0.0564 (P = 0.1069)	− 0.0437 (P = 0.8830)

^†^For regression analyses, the dependent variables (i.e., TBIL, AST, CRP) were normalized using the Box and Cox method.

### Correlation between Sano’s score for IVIG resistance and TTV viral load

**Figure 2:**
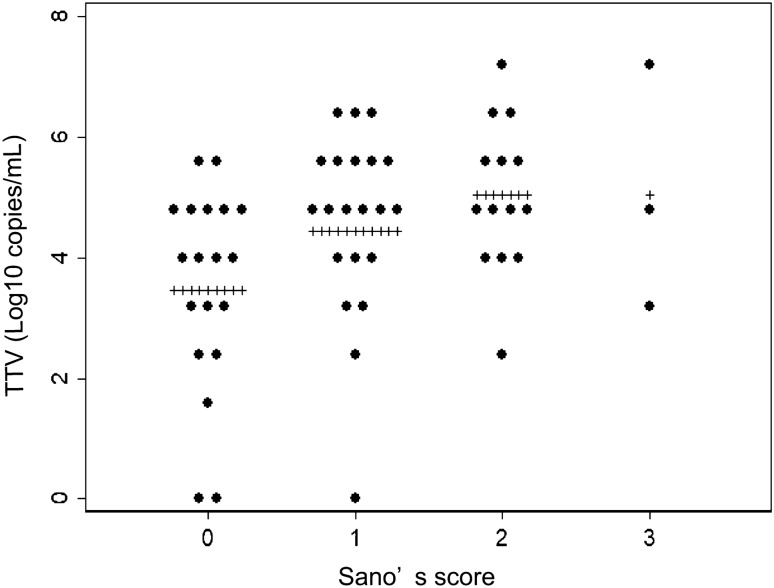
Sano’s score and TTV viral load. TTV viral loads in pre-treatment serum samples classified based on Sano’s score. Plus (+) indicates mean value in each group.

Sano’s scores were positively correlated with the TTV viral loads (Fig. 2). The statistical analyses indicated that Sano’s score was positively correlated with TTV viral load in Spearman’s rank correlation (R = 0.3645, P = 0.0045), a univariate logistic regression analysis (odds ratio [OR] 1.777, P = 0.0262), and a multivariate analysis that considered sex and age (OR 1.772, P = 0.0248) (Table 2).

**Table 2:** Logistic regression model to predict Sano’s score based upon Torquetenovirus viral loads and covariates.

n = 57	
(a) Univariate logistic regression analysis	Odds ratio (P value)
TTV	1.777 (P = 0.0262)

(b) Multivariate logistic regression analysis	Odds ratio (P value)
TTV	1.772 (P = 0.0248)
Sex (male: 1; female: 0)	0.622 (P = 0.4428)
Age (years)	0.929 (P = 0.6826)

### Correlation between Sano’s risk score and IVIG resistance

Resistance to the first IVIG treatment was correlated with Sano’s scores (Supplementary File 3). The correlation was statistically significant in a univariate analysis (OR 2.72, P = 0.0405. n = 50), and a multivariate regression analysis that considered age and sex (OR 3.05, P = 0.0366. n = 50).

### Correlation between TTV load and resistance to IVIG

**Figure 3:**
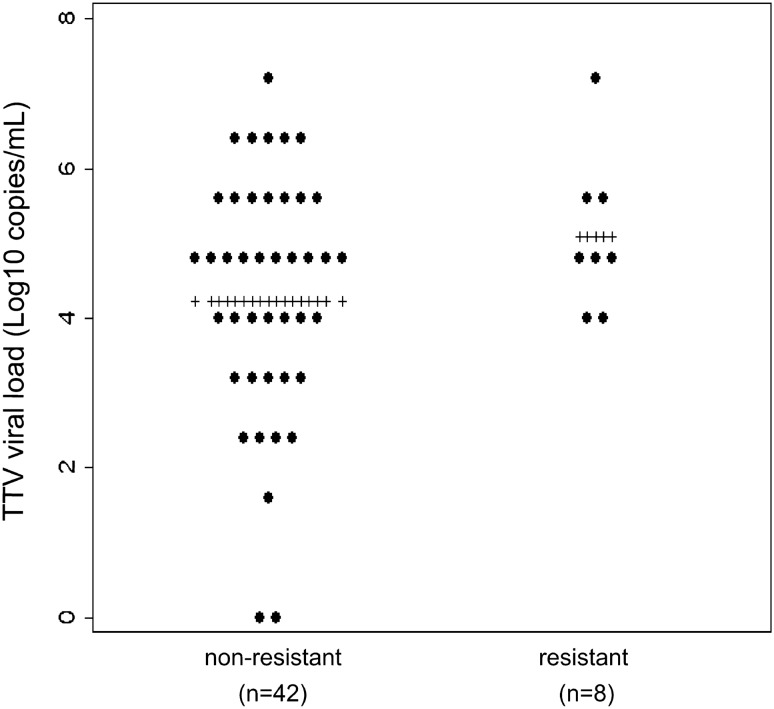
Resistance to intravenous immunoglobulin (IVIG) and TTV viral load. TTV viral loads in pre-treatment serum samples classified based on IVIG resistance. Plus (+) indicates mean value in each group.

In our participants, IVIG resistance had only a marginal correlation with TTV viral load in both the univariate and multivariate analyses (Fig. 3, Table 3). We used Monte Carlo simulation to estimate the sample size necessary to identify a statistically significant correlation (i.e., α = 0.05) between TTV load and IVIG resistance (Supplementary File 4). We considered ORs of 1 or 3 for male sex, and 1 or 0.7 for age (in years). Depending on the scenario, a sample size of 150–250 participants would achieve a statistical power of 80%.

**Table 3:** Logistic regression model to predict resistance to the first intravenous immunoglobulin, based on Torquetenovirus viral loads and covariates.

n = 50	
(a) Univariate logistic regression analysis	Odds ratio (P value)
TTV	1.569(P = 0.1567)

(b) Multivariate logistic regression analysis	Odds ratio (P value)
TTV	1.666 (P = 0.1544)
Sex (male: 1; female: 0)	3.652 (P = 0.1500)
Age (years)	1.135 (P = 0.5404)

### Temporal change in TTV viral load and IVIG resistance

**Figure 4:**
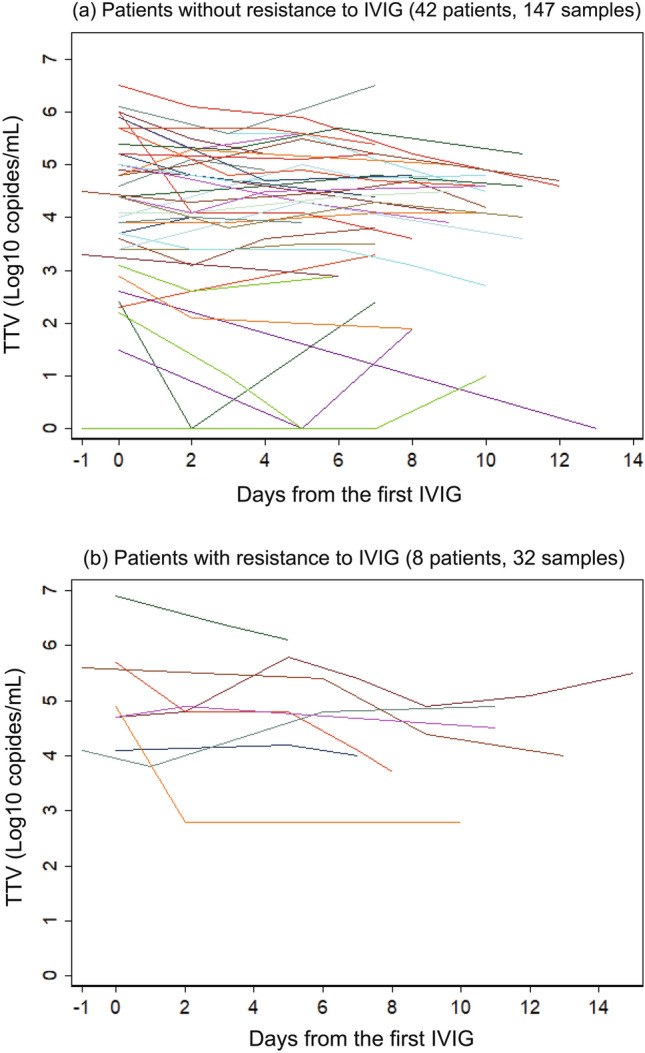
Temporal change in TTV viral load. TTV viral load versus days elapsed since day of first IVIG treatment for 42 patients (147 samples) who were not resistant (**a**), and 8 patients (32 samples) who were resistant (**b**) to IVIG.

The TTV viral load decreased over time (Fig. 4). In the entire population of participants, the viral load (log10 copies/mL) decreased by 0.042 per day (Table 4, column (a)). This result corresponded to a daily decrease of only 9.2%. This small downward trend was statistically significant (P = 0.0002) in the subgroup without IVIG resistance (Table 4, column (b)), but non-significant (P = 0.1304) in the group with IVIG resistance (Table 4, column (c)). However, the latter result may have been due to the small sample size available for this subgroup. We incorporated the presence of individual TTV species into this longitudinal analysis. However, due to the small sample size, none of the species showed any statistically significant contributions to the temporal trend of total TTV load. The dataset used for this longitudinal analysis is available in Supplementary File 5.

**Table 4:** Random effect regression analysis to explain Torquetenovirus viral loads.

	(a) All patients 50 patients 179 samples	(b) Patients without resistance 42 patients 147 samples	(c) Patients with resistance 8 patients 32 samples
Univariate analysis			
Days after 1st IVIG	− 0.0420 (P = 0.0001)	− 0.0437 (P = 0.0002)	− 0.0376 (P = 0.1304)
Multivariate analysis			
Days after 1st IVIG	− 0.0417 (P = 0.0001)	− 0.0433 (P = 0.0003)	− 0.0383 (P = 0.1241)
Sex (male: 1; female: 0)	0.455 (P = 0.3072)	0.243 (P = 0.6398)	1.038 (P = 0.1546)
Age (years)	− 0.0746 (P = 0.4988)	− 0.0852 (P = 0.5223)	− 0.0583 (P = 0.6637)

### Presence of individual TTV species

The DNA extracted from the pre-treatment samples (n = 57) were sequenced by the amplicon NGS technology (Accession number: PRJDB15714). Each of all 22 TTV species, which are defined in the update taxonomic classification^27^, were examined bioinformatically. Consequently, 17 TTV species were present in at least one pre-treatment samples (Supplementary File 6). Interestingly, the patient who presented the highest total TTV load and the largest TBIL value was positive only with TTV7 (Sample 402 in Supplementary File 1). The presence of TTV7 was marginally correlated with the normalised TBIL (Pearson’s R = 0.2172, P = 0.1046) as is shown in Supplementary File 6. The presence of TTV7 was positively correlated with the total TTV load (Pearson’s R = 0.3005, P = 0.0231, Supplementary File 6). TTV7 was positive in three KD patients (Supplementary File 7).

## Discussion

In this study, we found that the total viral load of TTV was associated with the hepatobiliary biomarkers that constitute Sano’s score^10^. A recent study in Japan indicated that TBIL has sensitivity and specificity which were superior to other biomarkers in predicting resistance to IVIG augmented by corticosteroid treatment^28^. Of note, all scores recommended in Japanese guidelines as being predictive for IVIG resistance (i.e., Kobayashi, Egami, and Sano) use liver enzyme(s)^10–12^. It has also long been assumed that KD severity is associated with hepatobiliary system damage^29,30^. Therefore, the significant correlation between TTV load and TBIL/AST identified in the present study suggested that TTV was involved in the hepatobiliary dysfunction which is crucial to the pathophysiology of KD. However, we are aware that the correlations between TTV load and the indicators of KD severity (i.e., Sano’s score, TBIL, and AST) identified in our present study do not necessarily demonstrate that there is a causal link between TTV and KD.

The correlation between TTV load and IVIG resistance was only marginally significant in the present study. This result might be due to the small sample size used. We estimated that 150–250 KD patients would be necessary to identify the presence of a statistically significant correlation between TTV load and IVIG resistance. However, the methylprednisolone pulse which was given to the high-risk patients (i.e. with ≥ 2 Sano scores) may have effectively prevented IVIG-resistance in some patients, and hence blunted the statistical power of our analysis. Therefore, a smaller sample size may be sufficient in a future study if the treatment to high-risk patients is not augmented with adjunct therapies including methylprednisolone pulse.

It is widely assumed that KD has an infectious trigger. Diverse microorganisms have been proposed as candidate causative agent(s) of KD^31,32^. Recently, it was reported that SARS-CoV-2 infection can result in an illness that mimics the symptoms and cardiac complications of KD^33^. Finding this illness, named paediatric inflammatory multisystem syndrome temporally associated with COVID-19^34^ or multisystem inflammatory syndrome in children (MIS-C)^35,36^, revived interest in a possible viral involvement in the pathophysiology of KD. However, to our knowledge, no study has found that a specific virus is associated with the development of KD or with illness severity.

In 1997, Nishizawa et al. first discovered TTV as a possible agent for hepatitis of unknown origin^37^. Subsequently, TTV was assigned to a new family, *Anelloviridae*^38^. During their lifetime, almost all human beings are infected with at least one TTV species that establishes a persistent infection^39^. TTV is associated with febrile episodes^40^, or respiratory illnesses^41,42^, in children. There are numerous reports of TTV involvement in liver diseases^43,44^. These results were consistent with our finding that a higher viral load of TTV was correlated with more severe hepatobiliary damage in patients with KD. The correlation between TTV load and hepatobiliary biomarkers for KD found in our study suggested that TTV plays a role in the pathophysiology of KD. Development of an experimental animal model would be crucial in understanding whether (and how) TTV is involved in the development of vasculitis, and in elucidating the relationship between TTV and KD.

Because TTV load is correlated with immunosuppression^45,46^, quantitative PCR of TTV has been established as a highly reliable and useful clinical methodology^25,47–49^. This methodology has been used in longitudinal studies^50,51^. Quantitative and longitudinal relationships between viral load and the course of illness are important in future pathophysiological studies of KD. Our study found that the TTV viral load decreased only slightly after the KD was treated with IVIG. However, it is unclear whether the temporal decrease in TTV viral load was due to the effect of IVIG because all patients received at least one IVIG dose. The temporal relationship between TTV viral load and IVIG resistance would be an interesting topic for future large-scale studies.

A newly developed TTV amplicon NGS showed that presence of TTV7 was marginally correlated with TBIL (P = 0.1046). This may be because TTV7 was significantly associated with larger total TTV loads (P = 0.0231). This finding is consistent with a previous report that the load of TTV7 in KD patients were high^24^. This hypothesis that TTV7 has a propensity of proliferating into a high viral load and hence of developing severe KD should be tested in a future study.

Mathematical studies predict that at least one agent for KD persistently infects the host^22,52^. Consistent with these predictions, TTV remains persistently in the host^39^. It was also predicted mathematically that the causative microbe for KD infects most children and that only a small proportion of these infections develop KD^22^. Almost all human beings are infected with at least one TTV species^43^. Therefore, TTV possesses many of the characteristics predicted for the agent(s) of KD. Although a previous study did not identify an association between TTV and KD^53^, we found that the PCR primer sets used in that study did not detect many species of TTV.

Our study had limitations, most importantly its small sample size and the absence of information for cardiac complications. In addition, our study lacked control subjects (i.e. children without KD). Although the fundamental pathophysiology of KD is systemic vasculitis, biomarkers for vasculitis (e.g. D-Dimer and Fibrin Degradation Products) were not recorded in our study. The objective of the original study, approved in 2001, was to elucidate the relationship between cytokine profiles and the severity of KD. Therefore, control subjects were not enrolled in this study. To address these issues, a future study should compare prevalence and viral load between patients with KD and control subjects. In this new study, indicators of vasculitis (e.g., D-Dimer and fibrin degradation products) should also be correlated to the TTV load.

In the Japanese diagnostic criteria for KD, the diagnosis of the complete form of KD is entertained when at least 5 signs/symptoms (e.g., fever, conjunctivitis, cheilitis, cervical lymphadenopathy, erythema and oedema of the fingers/toes) are present, regardless of the presence of overlapping infections^54^. Therefore, in the present study, KD was not diagnostically excluded solely based on the presence of infections. However, it would be interesting to consider not only TTV but many other microbes in a future study.

An advantage of our study was that our results were not interfered with COVID outbreak and MIS-C. A future large-scale study using updated methodologies is warranted to examine the relationship between TTV and KD.

## Conclusions

In 57 patients with KD, the viral load of TTV was positively correlated with the levels of hepatobiliary biomarkers constituting Sano’s score, which predicts resistance to IVIG in Japanese population. A larger study is necessary to identify a positive correlation between TTV and KD.

## Methods

### Ethical approval and informed consent

This study was approved by the ethics committee of the Osaka University Faculty of Medicine (references: 128-1 and 128-2) in November 2001. The guardians of the participants provided written informed consent to participate in the study. The samples were not used for the original objective and were stored at − 20 ℃. The samples were used for the present analysis in accordance with Japanese ethical guidelines for medical and biological sciences^55^.

### Diagnosis and treatment

KD was diagnosed based on Japanese diagnostic criteria, version 5^54^. All patients diagnosed as having KD were treated with IVIG (2 g/kg) and aspirin (30 mg/kg/day). An intravenous methylprednisolone pulse (30 mg/kg) was given to patients with KD who had values of 2 or 3 for Sano’s score, in parallel with the first IVIG. A patient who remained febrile (> 37.5 °C) 48 h after initiation of the first IVIG was classified as IVIG-resistant. A second IVIG treatment was given to all IVIG-resistant patients. When defervescence occurred, the dose of aspirin was decreased to 5 mg/kg/day. Blood tests were conducted before each IVIG, and at least once before discharge. A patient was discharged when the fever subsided for more than 48 h, all the biomarkers normalised and the coronary arteries did not show progressive dilatation.

### DNA extraction from serum

DNA was extracted from 200 μL serum using the QIAamp DNA Blood Mini Kit (Qiagen, Germany) following the manufacturer’s protocol. Extracted DNA was eluted into 200 μL buffer AE, which was then aliquoted in equal amounts into three tubes. The tubes were stored at – 80 °C until use.

### Quantification of total TTV DNA in individual samples

Extracted viral DNA was amplified using real-time PCR amplification performed on a 7500 Fast Instrument® (Applied Biosystems, MA, USA). The TTV load was determined using a single-step TaqMan real-time PCR assay that targeted the highly conserved 5’UTR of the TTV genome. Copy numbers were quantified as previously described^56,57^. Briefly, the method amplified a 63-nucleotide UTR fragment with a detection limit of < 10 viral genomes per ml plasma/serum.

### Amplicon NGS of TTV species

Targeted enrichment is often necessary to generate complete viral genomes in clinical samples^58^. For this aim, we developed an amplicon NGS method^59^, to identify individual TTV species. The sensitivity and specificity of this methodology will be reported elsewhere. We enriched the circular DNAs of anelloviruses, using Rolling Circle Amplification (RCA) technology which is based on Phi29 DNA polymerase^60^. RCA reaction was performed on a mixture of a DNA sample (< 10 ng), 25 µM of exonuclease-resistant random primer (Thermo Fisher Scientific, CA, USA), 4 mM of deoxynucleotides (dNTPs) (Solis BioDyne, Tartu, Estonia) and 10 U of φ29 DNA polymerase (Thermo Fisher Scientific, CA, USA). Amplification was performed at 30 °C for 18 h, followed by inactivation of Phi 29 DNA polymerase at 65 °C for 10 min. The resulting linear double-stranded DNA product was spectrophotometrically quantified by NanoDrop Lite instrument (Thermo Fisher Scientific, CA, USA) and tenfold diluted to be used as the template in the Universal Anelloviruses inverse-PCR^61^. The Universal Anellovirus inverse-PCR was applied to a mixture of 50 μL consisting of 1 × PrimeSTAR® GXL Buffer (Takara, Shiga, Japan), 200 μM dNTP mixture (Takara, Shiga, Japan), 0.3 μM forward primer, 0.3 μM reverse primer, 1.25 U of PrimeSTAR® GXL DNA polymerase (Takara, Shiga, Japan) and 500 to 1000 ng of template. Primers are as follows: 206INV_TTVFor, 5′-CAA GGG GCA ATT CGG GCT C-3′; 205INV_TTVRev, 5′-ACT NCG GTG TGT AAA CTC ACC T-3′. Positions of the 5′-end nucleotide of primers in the reference TTV1 genome (Accession: NC_002076) are used as primer prefix names with either forward (For) or reverse (Rev) orientation. The PCR was performed in 35 cycles of 98 °C for 10 s, 60 °C for 15 s, and 68 °C for 4 min. To verify successful amplification, 10 µL of product was analysed by electrophoresis on 1% w/v agarose gel. If the expected band was present on ultraviolet transilluminator, the remaining PCR product (about 40 µL) was purified by NucleoSpin Gel and PCR Clean-up (Macherey–Nagel, Düren, Germany). The purified PCR products were subjected to fluorimetric analysis using Qubit (Thermo Fisher Scientific, CA, USA) to determine the concentration of the recovered nucleic acids. Libraries were prepared using the Illumina DNA Prep kit (Illumina, San Diego, CA, USA), starting with an initial DNA input of 150 ng. The pool of libraries was quantified using Qubit, and the sizes of fragments were estimated using the BioAnalyzer (Agilent Technologies, Inc. CA, USA). The denatured libraries were then loaded in 12 pM concentration on Miseq V3 cartridge (Illumina, CA, USA) to be sequenced with Paired-end 2 × 250 bp.

### Bioinformatical analysis

We developed a bioinformatical pipeline which consists of reference-based approach and de-novo assembly. Briefly, low-quality reads were filtered and adapters were trimmed with Fastp programme^62^. Reads of human origin (Accession: GRCh38.p13) were eliminated using Kraken 2^63^. Using BBmap^64^, we aligned the remaining reads against a database of species-level anellovirus genomes^27^. The mapped reads reconstructed the full or near-full-length genomic sequences by consensus calling or de novo assembly. Subsequently, Blastn programme classified the reconstructed sequence into the anellovirus genomes at the species level. The reconstructed viral genomes were mapped with the non-human reads using BWA-MEM software version 0.7.17^65^. Then, the number of reads assigned to each individual species was reported in the log10 of reads mapped per million (Log RPM) (Supplementary File 1). We assumed that a TTV species was present in a sample when Log RPM was positive.

### Statistical analysis

Stata SE 13 (Stata, TX, USA) was used for the statistical analyses. In the linear regression analyses, the independent variables (i.e., TBIL, AST, and CRP) had highly skewed distributions. Therefore, these variables were normalised using the method proposed by Box and Cox^26^, as in the following equation:

$$\left[ {{\text{Normalised }}\;{\text{value}}} \right] \, = \, \left( {\left[ {{\text{original}}\;{\text{ value}}} \right]^{\lambda } - { 1 }} \right) \, / \, \lambda$$

“λ” was − 0.619589 for TBIL, − 0.5450522 for AST, and 0.4735706 for CRP. The results indicated that the normalised values had symmetrical distributions (Supplementary File 2).

### Sample size calculations

We estimated the sample size necessary to identify a statistically significant correlation between TTV viral load and IVIG resistance. We used Monte Carlo simulations^66,67^, and included sex and age as covariates in the multivariate logistic regression model. The distributions of viral loads and ages in our study participants were reproduced in the simulations. Age (years) was assumed to follow a two-parameter gamma distribution, with a shape parameter of 1.9 and a scale parameter of 1.5. Viral load (log10 copies per mL) was simulated as a normal distribution with a mean of 4.4 and a standard deviation of 1.55; the negative values were set to zero. The proportions of male and female participants were assumed to be equal. The OR for TTV viral load was fixed to 1.6, based on the results of the analysis (Table 3). The sensitivity analysis included an OR value for male sex of 1 or 3; the OR value for age (years) was 1 or 0.7, the latter of which was translated from a previously reported OR value (i.e., 0.974 for age in months)^11^. We conducted 10,000 simulations, and power was computed as the proportion of simulations in which the coefficient for viral load was statistically significant (i.e., two-sided P-value < 0.05). The Stata script for the simulation is available in Supplementary File 8.

### Supplementary Information

Supplementary Information 1.

Supplementary Information 2.

Supplementary Information 3.

Supplementary Information 4.

Supplementary Information 5.

Supplementary Information 6.

Supplementary Information 7.

Supplementary Information 8.

## Data Availability

All the data used in the present analysis are available as supplementary information. Nucleotide sequences obtained in the present study is registered in DNA Databank of Japan with a BioProject accession code of PRJDB15714 (BioSample: SAMD00598640-SAMD00598696).
