# Supplementary material for: Viral load of Torquetenovirus correlates with Sano’s score and levels of total bilirubin and aspartate aminotransferase in Kawasaki disease
Source: Sci Rep. 2023 Oct 21;13:18033. doi: 10.1038/s41598-023-45327-5 (PMC10590372; doi:10.1038/s41598-023-45327-5)
Supplement: Supplementary file 6 — Supplementary Information 6. [file 41598_2023_45327_MOESM6_ESM.doc]

| TTV | Number of positive patients (%) | TTV load | TBIL^†^ | AST^†^ | CRP^†^ |
| --- | --- | --- | --- | --- | --- |
| 1 | 4 (7%) | R=0.1708 (P=0.2039) | R=-0.0523 (P=0.6994) | R=-0.0866 (P=0.5217) | R=-0.0748 (P=0.5802) |
| 3 | 17 (30%) | R=0.3217 (P=0.0147*) | R=-0.0872 (P=0.5192) | R=-0.1008 (P=0.4556) | R=0.0062 (P=0.9635) |
| 4 | 1 (2%) | R=0.0682 (P=0.6143) | R=-0.0780 (P=0.5642) | R=-0.0688 (P=0.6111) | R=-0.1260 (P=0.3502) |
| 5 | 8 (14%) | R=0.2689 (P=0.0431*) | R=0.0633 (P=0.6402) | R=0.0413 (P=0.7603) | R=-0.0742 (P=0.5831) |
| 6 | 1 (2%) | R=0.0086 (P=0.9494) | R=-0.1975 (P=0.1409) | R=-0.1747 (P=0.1937) | R=-0.1099 (P=0.4158) |
| 7 | 3 (5%) | R=0.3005 (P=0.0231*) | R=0.2172 (P=0.1046) | R=0.1617 (P=0.2295) | R=-0.1025 (P=0.4481) |
| 9 | 13 (23%) | R=0.1801 (P=0.1800) | R=-0.0639 (P=0.6368) | R=-0.1157 (P=0.3913) | R=-0.1992 (P=0.1374) |
| 10 | 1 (2%) | R=0.0682 (P=0.6143) | R=-0.0780 (P=0.5642) | R=-0.0688 (P=0.6111) | R=-0.1260 (P=0.3502) |
| 13 | 6 (11%) | R=0.1477 (P=0.2729) | R=-0.0902 (P=0.5046) | R=-0.0825 (P=0.5419) | R=-0.0598 (P=0.6588) |
| 15 | 4 (7%) | R=0.1052 (P=0.4362) | R=0.0020 (P=0.9881) | R=0.0650 (P=0.6310) | R=0.0640 (P=0.6365) |
| 18 | 5 (9%) | R=0.1365 (P=0.3113) | R=-0.0273 (P=0.8401) | R=0.0011 (P=0.9937) | R=-0.1710 (P=0.2035) |
| 19 | 10 (18%) | R=0.1634 (P=0.2246) | R=-0.1179 (P=0.3824) | R=-0.2050 (P=0.1261) | R=-0.0525 (P=0.6981) |
| 20 | 5 (9%) | R=0.1819 (P=0.1756) | R=0.1405 (P=0.2971) | R=0.1068 (P=0.4291) | R=-0.2492 (P=0.0616) |
| 21 | 6 (11%) | R=0.1732 (P=0.1976) | R=-0.0978 (P=0.4690) | R=-0.2794 (P=0.0353) | R=0.1331 (P=0.3236) |
| 24 | 10 (18%) | R=0.2310 (P=0.0839) | R=-0.0271 (P=0.8415) | R=-0.0611 (P=0.6515) | R=0.1230 (P=0.3621) |
| 29 | 20 (35%) | R=0.1948 (P=0.1464) | R=-0.1165 (P=0.3882) | R=0.0817 (P=0.5456) | R=-0.0606 (P=0.6544) |
| 31 | 1 (2%) | R=0.1193 (P=0.3768) | R=0.1117 (P=0.4080) | R=-0.0964 (P=0.4755) | R=-0.0974 (P=0.4711) |

Pearson’s correlation coefficient (R) between the presence of individual TTV species, the total TTV load and the normalised biomarkers which predict resistance to intravenous immunoglobulin (n=57)

† TBIL, AST and CRP were transformed to normal distribution by Box & Cox method. * P<0.05
