# Supplementary material for: Viral load of Torquetenovirus correlates with Sano’s score and levels of total bilirubin and aspartate aminotransferase in Kawasaki disease
Source: Sci Rep. 2023 Oct 21;13:18033. doi: 10.1038/s41598-023-45327-5 (PMC10590372; doi:10.1038/s41598-023-45327-5)
Supplement: Supplementary file 7 — Supplementary Information 7. [file 41598_2023_45327_MOESM7_ESM.doc]

Three KD patients positive with TTV7

| Seq. in Supplementary File S1 | TBIL (mg/dL) | AST (IU/L) | CRP (mg/dL) | Sano’s score | TTV load  (Log10 copies/mL) |
| --- | --- | --- | --- | --- | --- |
| 95% CI† | 0.76−1.26 | 66−136 | 6.5−8.9 |  | 3.9−4.7 |
| 402 | 4.67 | 160 | 8.0 | 2 | 7.3 |
| 424 | 1.25 | 282 | 3.33 | 2 | 5.7 |
| 637 | 0.64 | 31 | 5.16 | 0 | 5.9 |

† 95% confidence interval (CI) in the entire patients (n=57).
